# A Warning to Married Men

**Published:** 1874-12

**Authors:** 


A Warning to Married Men.—A well-known manufacturer of this city visited his family a few days ago at one of the popular summer resorts not a thousand miles from Falmouth. Happening to have an unusually well-filled pocket-book, upon retiring for the night he placed it in one of his boots for safe keeping, omitting, however, to say anything about it to his wife. Fatigued with the long ride, he soon fell asleep, and, upon awakening early in the morning, sought in vain for his boots and his money. Rousing his wife with anxious inquiries about "those boots," he learned that she had found them lying around, and had set them outside the door for the boot-black. A few seconds later, a well-developed, manly form, with only "one or two clothes on," was noticed making rapid strides for the porter's lodge, with the "ragged edges of anxiety, remorse, rum, and despair" distinctly mapped out on his usually beaming countenance. It remains for us to add, as a simple act of justice to all parties, that the porter was honest, the money restored, and our manufacturer firmly convinced of the propriety of having no secrets from his wife in future.

